# Levodopa effects on [ ^11^C]raclopride binding in the resting human brain

**DOI:** 10.12688/f1000research.5672.1

**Published:** 2015-01-23

**Authors:** Kevin J. Black, Marilyn L. Piccirillo, Jonathan M. Koller, Tiffany Hseih, Lei Wang, Mark A. Mintun

**Affiliations:** 1 Departments of Psychiatry, Neurology, Radiology, and Anatomy & Neurobiology, Washington University School of Medicine, St. Louis, MO, 63110, USA; 2 School of Arts and Sciences, Washington University, St. Louis, MO, 63130, USA; 3 Department of Psychiatry, Washington University School of Medicine, St. Louis, MO, 63110, USA; 4 Departments of Psychiatry & Behavioral Sciences, and Radiology, Northwestern University Feinberg School of Medicine, Chicago, IL, 60611, USA; 5 Departments of Radiology, Psychiatry, Bioengineering, and Anatomy & Neurobiology, Washington University, St. Louis, MO, 63130, USA; 6 Temple University, Philadelphia, PA, USA; 7 Department of Ophthalmology, University of Cincinnati, Cincinnati, OH, USA; 8 Avid Radiopharmaceuticals, Philadelphia, PA, USA

**Keywords:** dopamine, D2, receptor, raclopride, positron, emission, tomography, PET, levodopa, dopamine, Tourette, syndrome, nucleus, accumbens, substantia, nigra, midbrain

## Abstract

**Rationale:** Synaptic dopamine (DA) release induced by amphetamine or other experimental manipulations can displace [ ^11^C]raclopride (RAC*) from dopamine D2-like receptors. We hypothesized that exogenous levodopa might increase dopamine release at striatal synapses under some conditions but not others, allowing a more naturalistic assessment of presynaptic dopaminergic function. Presynaptic dopaminergic abnormalities have been reported in Tourette syndrome (TS).

**Objective:** Test whether levodopa induces measurable synaptic DA release in healthy people at rest, and gather pilot data in TS.

**Methods:** This double-blind crossover study used RAC* and positron emission tomography (PET) to measure synaptic dopamine release 4 times in each of 10 carbidopa-pretreated, neuroleptic-naïve adults: before and during an infusion of levodopa on one day and placebo on another (in random order). Five subjects had TS and 5 were matched controls. RAC* binding potential (BP _ND_) was quantified in predefined anatomical volumes of interest (VOIs). A separate analysis compared BP _ND_ voxel by voxel over the entire brain.

**Results:** DA release declined between the first and second scan of each day (p=0.012), including on the placebo day. Levodopa did not significantly reduce striatal RAC* binding and striatal binding did not differ significantly between TS and control groups. However, levodopa’s effect on DA release differed significantly in a right midbrain region (p=0.002, corrected), where levodopa displaced RAC* by 59% in control subjects but *increased* BP _ND_ by 74% in TS subjects.

**Discussion:** Decreased DA release on the second scan of the day is consistent with the few previous studies with a similar design, and may indicate habituation to study procedures. We hypothesize that mesostriatal DA neurons fire relatively little while subjects rest, possibly explaining the non-significant effect of levodopa on striatal RAC* binding. The modest sample size argues for caution in interpreting the group difference in midbrain DA release with levodopa.

## Introduction

Dopamine (DA) release from neurons has often been conceptualized as occurring via two separable mechanisms: tonic, referring to low levels of DA in extrasynaptic spaces that may be more accessible to microdialysis, and phasic, referring to synaptic DA release at synapses following presynaptic action potentials ^1^. Phasic dopamine release is crucial to dopamine’s role in changing behavior ^2^, including in learning sequences of movements ^3^. Normal tonic dopamine release but abnormal phasic dopamine release has been postulated to occur in several disease states, including drug abuse ^4^ and Tourette syndrome (TS) ^5– 8^.

The radioligand [ ^11^C]raclopride (hereinafter RAC*) binds to dopamine D _2_-like (D _2_, D _3_ and D _4_) receptors loosely enough to be displaced by physiological increases of dopamine at the synapse. This property has been exploited to detect changes in synaptic DA release induced by experimental manipulations including the administration of amphetamine ^9^. However, amphetamine also has some disadvantages in this context—primarily, that it does not really produce *phasic* dopamine release in the usual, temporal, sense of the word. Rather, it causes prolonged, substantial dopamine release regardless of environmental demands. Scientific questions about DA release in the absence of amphetamine might be better tested with a pharmacological stimulus that could potentially increase the magnitude of DA release, but under tighter endogenous control. Additionally, amphetamine can induce symptomatic effects including euphoria ^10^ and transiently increased tic severity ^11^; these effects can themselves alter brain activity, complicating interpretation of the results. Ideally, a pharmacological challenge drug to test phasic dopamine release would not produce effects noticed by the subject.

The present study provides preliminary data for a novel approach to testing presynaptic dopamine release using levodopa, the body’s natural synthetic precursor to dopamine. Exogenous levodopa boosts dopamine synthesis almost immediately in both parkinsonian and healthy brains [reviewed in 12]. The extra dopamine is rapidly released at the synapse in people with DA deficiency ^13^, and there is evidence that this happens also in the non-parkinsonian brain. In people, including in people with tics, levodopa produces dose-dependent yawning, mild sleepiness, and effects on working memory— *i.e.*, CNS-mediated effects ^14– 16^. Additional evidence for levodopa-induced synaptic DA release in the non-parkinsonian brain is reviewed in 12. When given after an adequate dose of carbidopa, which prevents conversion to dopamine but does not cross the blood-brain barrier, systemic levodopa administration essentially delivers dopamine selectively to the brain, as confirmed by the fact that it does not alter quantitative whole-brain blood flow ^17– 19^, as dopamine would if it were being delivered systemically or produced outside the brain. In fact, with adequate carbidopa pretreatment, volunteers usually cannot tell whether they are receiving levodopa or a placebo ^12, 16^.

We used PET and RAC* to measure synaptic dopamine release in response to a standardized levodopa infusion (after carbidopa) in 10 subjects. Since no previous data were available on levodopa effects on RAC* PET, we included before- and during-levodopa RAC* PET scans as well as before- and during-placebo scans. Half of the subjects had a chronic tic disorder and the other half were matched control subjects without tics, to generate preliminary data in each population. The original hypotheses were that levodopa would stimulate striatal dopamine production in the controls, but may affect people with TS differently.

## Methods

### Participants

This study was approved by the Human Studies Committee of Washington University School of Medicine (IRB, protocol # 03-0347, the WUSM Radioactive Drug Research Committee (protocol # 497F), and the U.S. Food and Drug Administration (Investigator IND #69,745 for i.v. levodopa). All subjects provided written confirmation of informed consent before study participation.

Diagnostic assessment included psychiatric and neurological examination by a movement-disorders-trained neuropsychiatrist (KJB) and a validated semistandardized psychiatric diagnostic interview [SCID-IV; 20]. Tic subjects met DSM-IV-TR criteria for Tourette’s Disorder. Control subjects with no history of tics were matched one-to-one for age, sex and handedness (with one ambidextrous TS subject matched to a right-handed control). Exclusion criteria included any lifetime neurological or Axis I psychiatric disorder (except that TS, ADHD and OCD were allowed in tic subjects, and migraine and specific phobia were allowed in either group), current serious general medical illness, medication history of dopamine antagonists or other drugs likely to affect the dopaminergic system, current use of any neuroactive medication, lactation, possibility of pregnancy, or contraindication to levodopa or MRI.

Clinical features were characterized by the Diagnostic Confidence Index (0=no features of TS; 100=all enumerated features of classic TS; scores in the original clinical validation sample ranged from 5 to 100, mean=61, S.D.=20) ^21^; the YGTSS, an expert-rated measure of tic severity over the previous week (motor tic scale 0–25, vocal tic scale 0–25, impairment scale 0–50, higher scores indicating a higher symptom burden) ^22, 23^; the revised Tic Symptom Self-Report (TSSR) scale, a self-report scale including scores of 0–3 for each of 18 motor tics and 16 vocal tics, with 3 indicating tics were “very frequent and very forceful” over the preceding two weeks ^24, 25^; the ADHD Rating Scale, an expert-rated measure of current severity of Attention-Deficit/Hyperactivity Disorder (ADHD) based on DSM-IV criteria (range 0–54, higher scores indicating a higher symptom burden) ^26^; and the Y BOCS, an expert-rated measure of current obsessive-compulsive disorder (OCD) severity (range 0–40, higher scores indicating a higher symptom burden) ^27, 28^.

### Overview of subject participation

**Figure 1.**
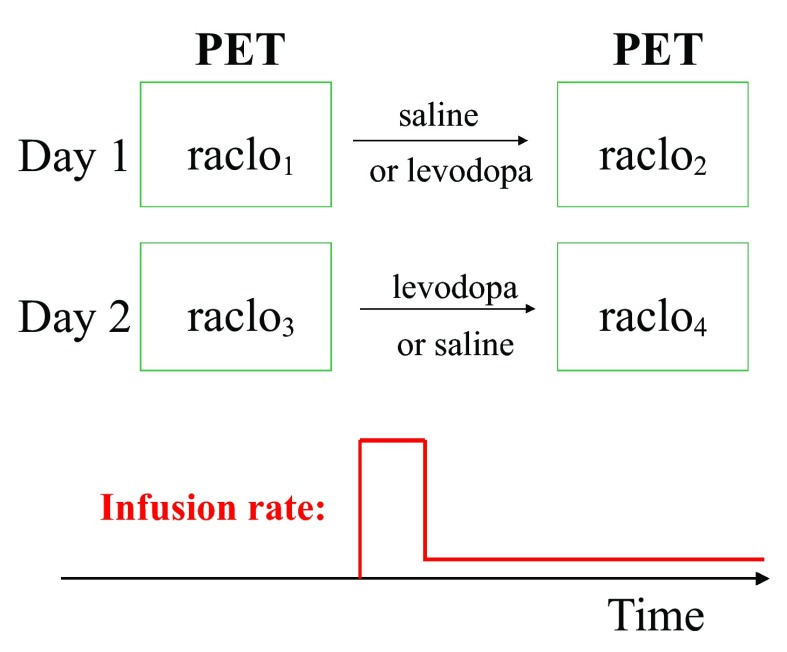
Study overview.

Each subject had 4 RAC* PET scans: two scans on each of two days at least a week apart ( Figure 1). After oral carbidopa and the baseline PET scan, an infusion of levodopa or saline placebo was begun by vein at an individualized dose intended to produce a steady-state levodopa plasma concentration of 600ng/mL. After allowing 30 minutes to approach steady-state levodopa concentration, a second scan was done while the infusion continued. The order (levodopa on day 1 and placebo on day 2, or the reverse) was assigned randomly to each subject, and subjects and PET staff were blind to drug assignment during all scans.

The room was darkened and subjects were instructed to lie quietly in the scanner with eyes closed throughout each scan. Study staff asked subjects every 5 or 10 minutes if they were comfortable and made sure they were awake.

### Levodopa infusion

Subjects took 200mg carbidopa by mouth at least 1 hour before levodopa infusion began. A dose of levodopa estimated to fill each subject’s volume of distribution at a target concentration of 600ng/mL was infused over 10 minutes, followed until the second PET scan of the day was completed by a maintenance infusion at a rate estimated to compensate for elimination. In prior work, these infusion rates produced a mean blood level across subjects of ~625ng/mL after 25 minutes of infusion ^16^. On average, that concentration produces substantial motor benefit in early Parkinson disease ^29, 30^, yet this infusion method is well enough tolerated that subjects cannot reliably distinguish the levodopa and saline infusions ^12, 16^.

### Levodopa plasma concentration

Levodopa plasma concentration was measured by a validated method ^31^.

### Radiotracer preparation

[ ^11^C]raclopride was prepared by *O*-[ ^11^C]methylation of (S)- *O*-desmethylraclopride HBr (ABX Advanced Biochemical Compounds, Radeberg, Germany) using a modification of previously reported procedures ^32, 33^. Carbon-11 was produced as ^11^CO _2_ using the Washington University JSW BC 16/8 cyclotron and the ^14^N(p,α) ^11^C nuclear reaction. The ^11^CO _2_ was converted to ^11^CH _3_I using the microprocessor-controlled PETtrace MeI MicroLab (GE Medical Systems, Milwaukee, WI), and immediately used for [ ^11^C]methylation of (S)- *O-*desmethylraclopride. Product [ ^11^C]raclopride was purified via semipreparative HPLC, and reformulated in a 10% ethanol/normal saline solution. The radiochemical purity exceeded 95%, and the specific activity exceeded 500 Ci/mmol, as determined by analytical HPLC. The mass of raclopride was ≤13.9 µg per injected dose.

### Image acquisition

RAC* was given i.v. over an interval of 30 seconds (median dose 14.8mCi, interquartile range 11.0–18.9mCi). PET images were acquired on a Siemens ECAT 961 camera beginning with arrival of radiotracer in the head and continuing for 60 minutes using image frames of increasing duration. An MP-RAGE sequence was used to acquire a 3-dimensional T1-weighted image of the brain with acquisition time ~400 sec and voxel dimensions 1.25×1×1mm ^3^.

### Image alignment

The PET images were realigned within each subject and then to the subject’s MRI using a rigid-body alignment method with low measured error, optimized for dynamic PET images ^34– 37^.

### VOI analysis

**Figure 2.**
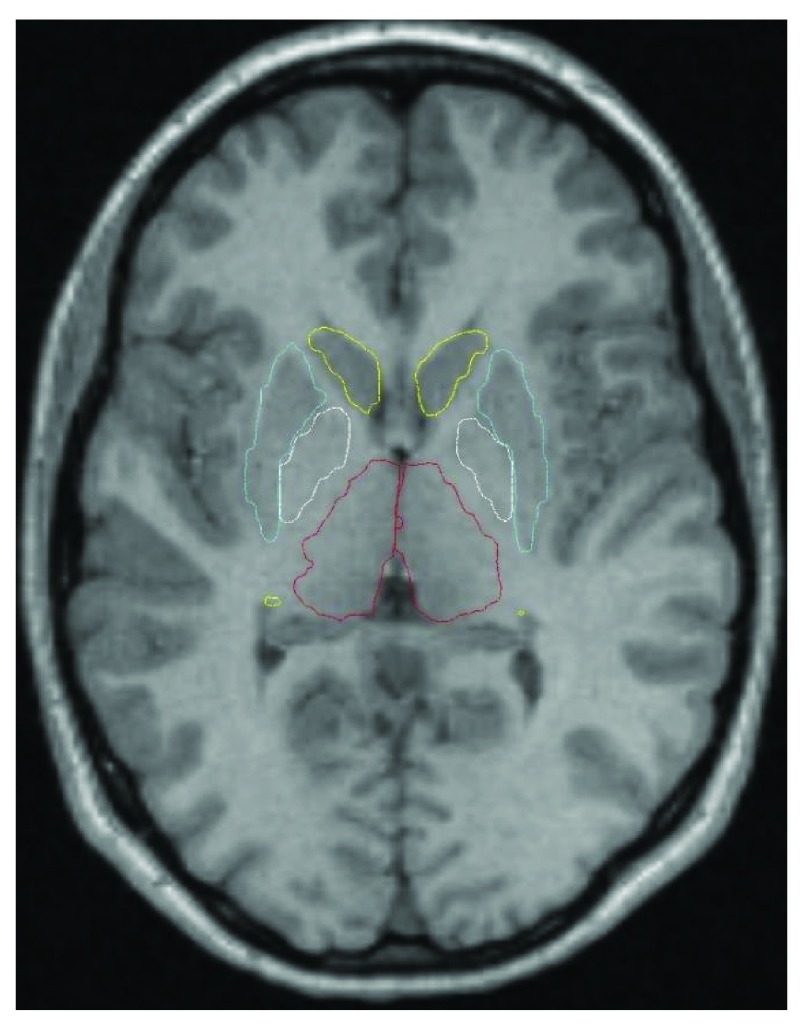
Automated striatal VOIs. Atlas-based VOI outlines are shown on an axial section from one subject (Cd yellow, Pu light blue, Pl white, Th red; NA does not appear on this section).

**Figure 3.**
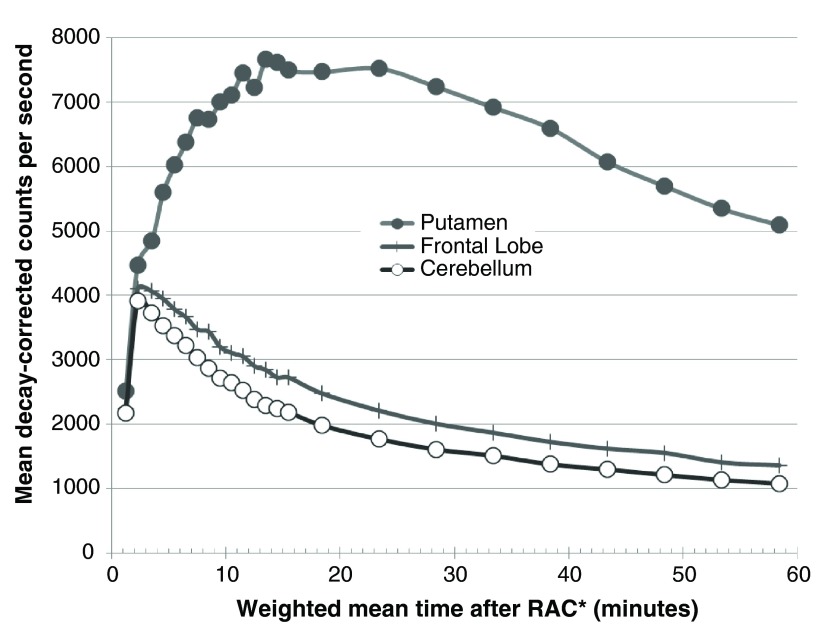
Time-activity curves. Decay-corrected time-activity curves are shown for the right putamen (filled circles), the frontal lobe VOI (+’s), and the cerebellar reference region (empty circles) from one subject’s pre-levodopa PET scan.

Nine subcortical volumes of interest (VOIs) were defined for each subject from that subject’s MRI by a high-dimensional semi-automated method of known high test-retest reliability ^38^ ( Figure 2). These VOIs corresponded to the thalamus and the left and right putamen, caudate, nucleus accumbens, and globus pallidus. An additional VOI was created from the average (weighted by region volume) of 22 FreeSurfer-labeled gray matter regions comprising frontal cortex (11 left- and 11 right-hemisphere VOIs). This large frontal VOI produced adequate counting statistics for modest noise in the time-activity curve ( Figure 3). A cerebellum VOI was traced on each subject’s MR image. All VOIs were transferred to each subject’s realigned PET images using the optimized MRI-to-PET transformation matrix computed in the alignment step. The cerebellar VOI was trimmed if needed so that no voxel in the VOI corresponded to any of the inferior-most four slices in any frame of that subject’s original PET images. Thus in each subject each VOI was identical for all four PET scans.

The binding potential BP _ND_^39, 40^, an estimate of the quotient B _max_/K _D_, was computed as one less than the distribution volume ratio (DVR), which was derived for each of the nine subcortical VOIs and the frontal lobe VOI using the cerebellar reference region ^41^. As we had no *a priori* hypothesis about laterality of results in any of the paired basal ganglia nuclei, we averaged corresponding left and right BP _ND_s (weighted by VOI volume) to produce for each PET scan six final BP _ND_ values, one each for frontal lobe cortex (FL), thalamus (Th), putamen (Pu), caudate (Cd), nucleus accumbens (NA), and globus pallidus (Pl).

The primary statistical analysis used a repeated-measures analysis of variance (rmANOVA) with BP _ND_ as dependent variable, diagnosis (tic or control) as a between-group variable, time (before or during the infusion) and day (placebo or levodopa) as within-subject variables, and region (the six VOI-based BP _ND_s) as a repeated measure. Exploratory analyses used an ANOVA for each region.

### Whole-brain analysis

For each subject, a DVR image was computed using at each voxel in the brain the Logan graphical method with the cerebellar VOI described in the preceding section as reference region ^41^. As a methods check, the mean across striatal VOIs of the voxelwise DVR value was essentially identical to the regional DVR computed using the standard methods described above. Analysis was limited to voxels in atlas space at which every subject contributed data from all frames of the dynamic PET acquisition.

Whole-brain comparisons used voxelwise *t* tests corrected for multiple comparisons in SPM 8, as follows. A *t* test compared DVR images between the TS and the control group, and clusters of contiguous voxels with *t* exceeding the threshold corresponding to *p*<0.001 were accepted as significantly different between groups if cluster volume exceeded the threshold required to control False Discovery Rate (FDR) for the entire dataset at *p*<0.05.

Two comparisons were made, one based on mean baseline DVR images and the other based on levodopa effect ΔDVR images. Each subject’s two pre-infusion RAC* PET scans, one from each scan day, were averaged to create that subject’s mean baseline DVR image. The difference of the during-levodopa DVR image and the during-placebo DVR image in a subject was used to create that subject’s levodopa effect ΔDVR image.

## Results

### Subjects

Subject characteristics and adequacy of matching are reported in Table 1, and clinical characteristics of the TS group are reported in Table 2.

**Table 1.** Subject characteristics and adequacy of matching.

Measure	Tic Subjects (N=5)	Controls (N=5)
Age (years; mean ± S.D.)	33.8 ± 12.9	32.8 ± 11.1
Sex, male (N)	4	4
Race, Caucasian (N)	4	4
Handedness, right (N)	4	3
OCD diagnosis (N)	1	0
ADHD diagnosis (N)	2	0

Abbreviations: OCD=Obsessive-compulsive disorder, ADHD=Attention Deficit Hyperactivity Disorder.

**Table 2.** Clinical characteristics of the Tourette syndrome group. The Y BOCS was completed for only 1 tic subject; the score was 9 on day 1 and 14 on day 2

Scale		Scores (mean ± S.D.)
DCI score		36.8 ± 22.0
YGTSS	Motor tic score	10.6 ± 3.4
	Vocal tic score	7.8 ± 4.0
	Impairment score	9.4 ± 9.8
TSSR score	Motor	9.3 ± 5.9
	Vocal	3.2 ± 2.3
	Total	12.5 ± 7.9
ADHD Rating Scale		11.6 ± 10.7

Abbreviations: DCI=Tourette Syndrome Diagnostic Confidence Index, YGTSS=Yale Global Tic Severity Scale, Y-BOCS=Yale-Brown Obsessive Compulsive Scale, ADHD=Attention Deficit Hyperactivity Disorder, TSSR=Tic Symptom Self Report.

### Levodopa levels

Levodopa plasma concentrations were ~800–1000ng/ml before the RAC* scan and ~500–700ng/ml after the RAC* scan, and did not differ significantly between groups ( Table 3).

**Table 3.** Levodopa plasma concentrations in ng/ml, mean ± SD.

Time	Controls	Tic subjects	*p* ( *t* test)
Peak (10' into infusion)	1591.5 ± 232.5	1938.8 ± 726.3	0.36
Just before RAC* scan	788.0 ± 152.4	992.4 ± 322.9	0.26
Just after RAC* scan	529.5 ± 149.2	662.8 ± 136.1	0.21

### Counting statistics in VOIs at baseline

**Figure 4.**
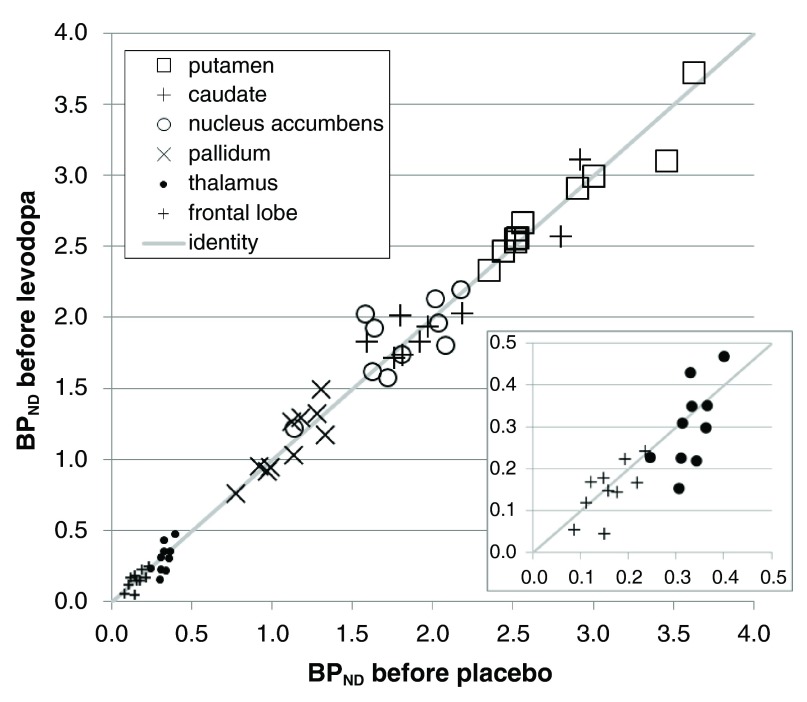
Stability of baseline binding between scan days in the *a priori* VOIs. BP _ND_s from the first scan of each day are plotted for all 10 subjects, with the BP _ND_ from the pre-placebo scan on the horizontal axis and from the pre-levodopa scan on the vertical axis. For the paired VOIs the mean of the left and right BP _ND_ is used. The diagonal line is the line of identity. The inset shows an enlarged view of the data from the frontal lobe and thalamus VOIs.

The *a priori* VOIs showed higher and more reliable binding in striatum and pallidum, as expected. Nevertheless, the thalamus, GP and frontal cortex VOIs also produced good counting statistics ( Figure 3). For every one of the VOIs the baseline BP _ND_ estimates were positive in all 120 scans, and were very similar between the two scan days ( Table 4, Figure 4).

**Table 4.** RAC* binding in *a priori* VOIs at baseline.

VOI	FL	Th	Pl	NA	Cd	Pu
BP _ND_ (mean)	0.15	0.32	1.11	1.80	2.13	2.79
BP _ND_ (standard deviation)	0.05	0.08	0.20	0.30	0.45	0.42
BP _ND_ values > 0 (of 20 scans)	20	20	20	20	20	20
*p* for mean > 0 (one-sample *t* test)	.0000	.0000	.0000	.0000	.0000	.0000
Correlation *r* between days, across subjects	.70	.63	.88	.76	.94	.96
*p* for correlation (8 df, 1 tail)	.012	.025	.0003	.005	.0000	.0000

Abbreviations: FL, frontal lobes; Th, thalamus; Pl, pallidum; NA, nucleus accumbens; Cd, caudate; Pu, putamen.

### Stability of RAC* binding between days and with time

This study includes a before- and after-infusion scan on each of two days. On one day the infusion contains levodopa, and on the other day it is a saline placebo. Thus each subject has three non-levodopa scans (the first scan of each day plus the scan during the placebo infusion). As expected, BP _ND_ was quite reproducible in the two pre-levodopa scans (correlated at *r* = 0.99 across VOI and subject).

**Figure 5.**
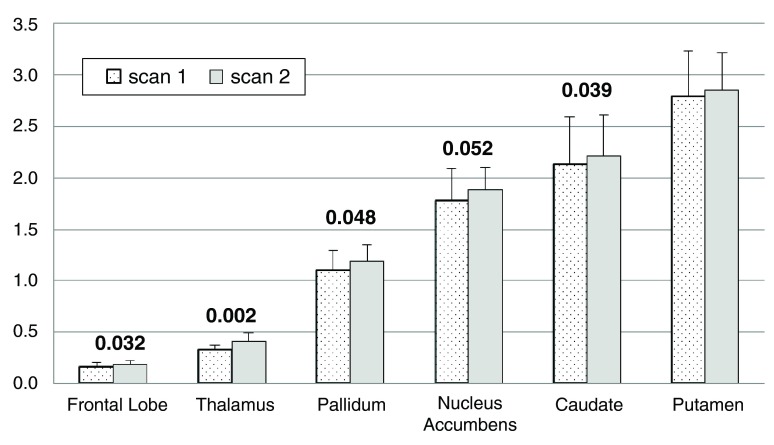
Change in BP _ND_ on the placebo day. For each of the *a priori* VOIs, mean BP _ND_ across all 10 subjects is shown before and during the infusion on the placebo day only. Error bars show SD. Numeric labels are *p* values for the main effect of time in the individual region ANOVAs (putamen *p*=.115).

To our surprise, BP _ND_ increased between the 1 ^st^ and 2 ^nd^ scan of the day (main effect of time, F=10.605, df=1,8, p=0.012), and this change did not differ significantly between the levodopa and placebo days (time × day interaction, F=0.014, df=5,4, p=0.909). In other words, the two scans on the placebo day were *not* identical. Mean BP _ND_ was 2.7% to 24.0% higher during the *placebo* infusion, indicating decreased dopamine release compared to earlier on the same day. The change from the first to the second scan of each day was significant in most individual region analyses: main effect of time, thalamus p=0.002, frontal lobe p=0.032, caudate p=0.039, pallidum p=0.048, and nucleus accumbens p=0.052 (multivariate time × region interaction F=4.173, df=5,4, p=0.096). Figure 5 shows the BP _ND_ for each VOI from both scans on the placebo day only.

### Effect of levodopa on RAC* binding

Since the pre- and on-placebo scans differed, the appropriate comparison for the on-levodopa RAC* scan is the on-placebo scan. Therefore we assessed the effect of levodopa by comparing the BP _ND _in the on-LD and on-placebo scans. In the VOI analysis, there was no significant effect of LD (day × time interaction, F=0.014, df=1,8, p=0.909).

### Comparison of RAC* binding between TS and control groups

***TS vs control at baseline.*** For the ANCOVA across all regions, RAC* binding did not differ significantly between tic and control subjects (main effect of diagnosis, F=0.744, df=1,8, p=0.413; tic vs control). Nevertheless, baseline RAC* binding was numerically higher in TS by 13–17% in the three striatal VOIs and by 5–7% in the frontal lobe and thalamus VOIs. The whole-brain analysis identified no significant regional differences in baseline RAC* binding between TS and control subjects.

**Figure 6.**
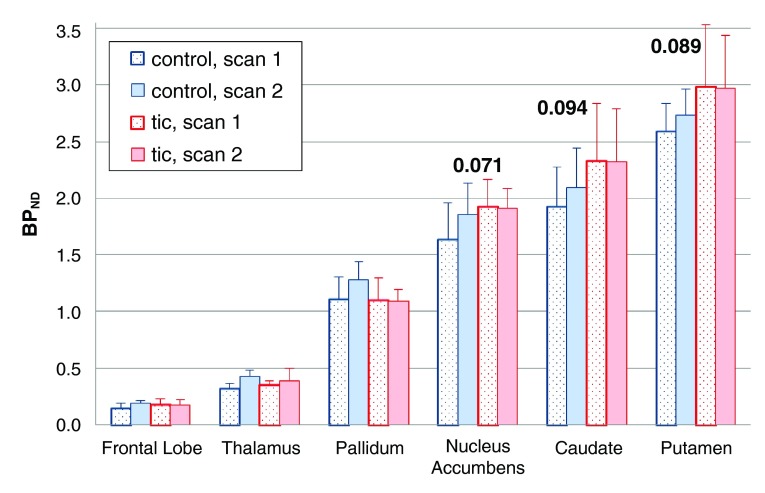
Change in [ ^11^C]raclopride binding on placebo day, by diagnosis. Mean BP _ND_s from the *a priori* VOIs, before and during the infusion on the placebo day only. Error bars show SD. The *p* values shown are for the time × diagnosis interaction in the individual region ANOVAs.

***TS vs control: time effect (change from first to second scan).*** There was a trend for the change in BP _ND_ during the infusion to be smaller in tic subjects (time × diagnosis interaction F=4.211, df=1,8, p=0.074). Each of the three striatal regions showed a similar effect when analyzed individually (0.05 < p < 0.10). Figure 6 shows the VOI BP _ND_ values before and during the placebo infusion, by diagnosis.

**Figure 7.**
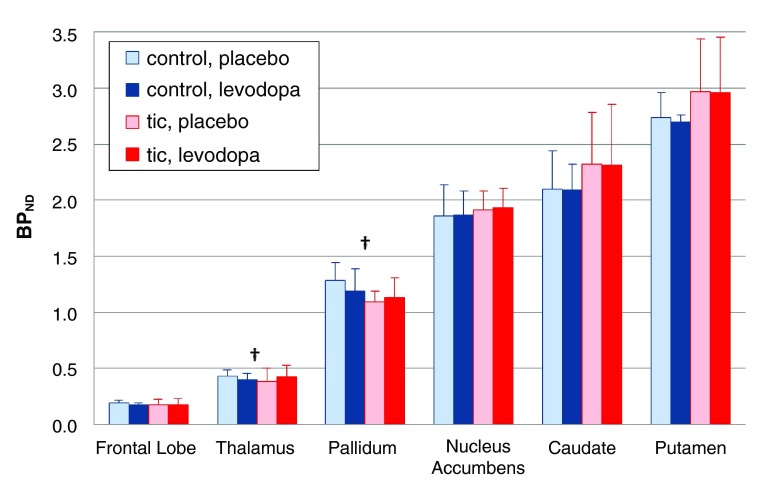
Levodopa-induced change in BP _ND_, by diagnosis. Mean BP _ND_ for the *a priori* VOIs is shown during the levodopa and placebo infusions; the error bar indicates SD. The day × time × diagnosis interaction and the day × time × diagnosis × region interaction were not significant. The daggers indicate a trend in the thalamic and pallidal VOIs for BP _ND_ to decrease with levodopa in the control group but increase with levodopa in the tic group (regional ANOVA, day × time × diagnosis interaction, pallidum p=0.050, thalamus p=0.098).

TS vs control: effect of levodopa on RAC* binding. In the *a priori* VOIs, the effect of LD did not differ overall in tic subjects (day × time × diagnosis interaction, F=1.308, df=1,8, p=0.286), and the 4-way interaction (day × time × diagnosis × region) was not significant (F=1.577, df=5,4, p=0.340). Although not statistically significant, pallidal and thalamic BP _ND_ tended to decrease in control subjects but increase in the tic subjects ( Figure 7).

**Figure 8.**
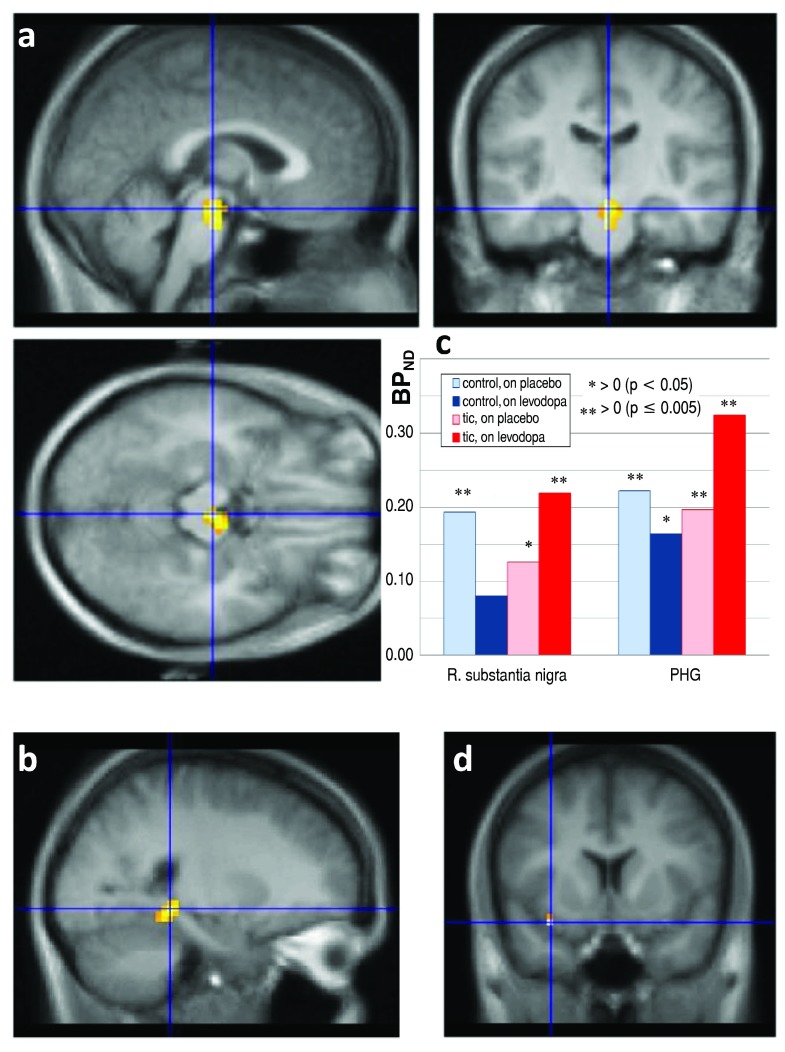
RAC* binding on levodopa vs. placebo, by diagnosis. Differences in the RAC* binding response to levodopa between TS and control subjects, thresholded at uncorrected *p* = 0.001, in color, laid over the MRI template image in grayscale. **a**, **b**: Significant clusters, with blue lines crossing at the peak *t* value in midbrain ( **a**, three views) and in parahippocampal gyrus ( **b**). A third statistically significant cluster was centered at the posterior edge of the occipital lobe, but both the location and the observation that in this cluster the BP _ND_ on placebo was negative in half the subjects suggests that this cluster likely does not reflect specific binding. **c**: Levodopa-induced change in BP _ND_, TS vs. control, in the clusters shown in A and B. R., Right; PHG, parahippocampal gyrus. Asterisks indicate that mean BP _ND_ differs significantly from zero. **d**: The blue lines cross at the voxel with the highest t value in the whole-brain SPM analysis of levodopa effect ΔDVR images ( *t*=11.62, 8 df).

The whole-brain analysis identified a similar but statistically significant effect in two clusters, where RAC* binding decreased with levodopa in controls, consistent with increased dopamine release during the levodopa infusion, but RAC* binding increased in the TS group. The first cluster included 38 voxels in midbrain (1.0 ml, FDR corrected *p*=0.002), with a peak *t* value of 9.0 (8 df) at atlas coordinate (1.5, −21, −15) and extending laterally in approximately the right substantia nigra/ventral tegmental area ( Figure 8a). A second significant cluster of 19 voxels (0.5 ml, corrected *p*=0.023) occurred in parahippocampal gyrus, with peak *t*=7.92 at (22.5, −39, −6) ( Figure 8b). The mean change in BP _ND_ with levodopa in these regions is shown in Figure 8c. In both these clusters, the BP _ND_ on placebo was positive in all subjects ( *p* < 0.001, binomial distribution), consistent with nontrivial RAC* binding. The highest *t* value in the whole-brain comparison, 11.62, occurred in Brodmann’s area 13, but the cluster volume was only 0.1 ml, not significant by FDR correction ( Figure 8d).

## Discussion

### Change in striatal BP _ND_ on the placebo day

BP _ND_ increased from before to during the placebo infusion in the striatum, thalamus and frontal lobe VOIs, especially in control subjects ( Figure 5, Figure 6). Surprisingly little information describes within-day stability of RAC* binding, though several studies compare binding across time intervals of days to months ^42– 45^. Mawlawi *et al.* ^46^ scanned 10 subjects twice each on the same day using a bolus-plus-constant-infusion method, and found no significant mean change from the first to the second scan. However, Alakurtti and colleagues ^47^ found that mean BP _ND_ increased from the first to the second scan of the day in striatal and thalamic regions, with the change (about +5%) reaching statistical significance in medial and lateral thalamus.

The observation in the present study that BP _ND_ increased from the first to second scan of the day is consistent with this background, and is relevant to RAC* challenge PET studies in general, because essentially all such studies use a before- *vs.* after-intervention design. Slifstein *et al.* [ 48, p. 357] argue that the existence of placebo-induced DA responses make the before-after model more appropriate for amphetamine challenge studies. However, our results and those of Alakurtti *et al.* ^47^ suggest that BP _ND_ increases from the first to the second scan even without active intervention. This does not invalidate the results of most before-after RAC* studies, since amphetamine challenge *decreases* striatal RAC* BP _ND_ by a large fraction, and to a lesser extent so do many cognitive and behavioral interventions in such studies, including studies of the placebo effect. However, the present results suggest that before-after RAC* studies may be less sensitive to manipulations that would decrease dopamine release.

***Possible pathophysiological interpretation.*** The increase in BP _ND_ during the placebo infusion is most likely associated with passage of time rather than a placebo effect *per se*, especially as placebo administration is more likely to increase dopamine release ^48– 50^. The presumed decrease in dopamine release during the placebo infusion could indicate that control subjects accommodate to the scanner environment over the course of the study day.

### Effect of levodopa infusion on RAC* binding

Levodopa effect on RAC* binding in striatum. Striatal RAC* binding was not substantially changed by levodopa. Initially this result came as a surprise to the authors, because levodopa was given expressly with the expectation that it would increase synaptic dopamine levels. Briefly, support for this expectation includes the following. First, in Parkinson disease there is overwhelming evidence both by clinical observations and by RAC* PET imaging that exogenous levodopa substantially increases striatal dopamine release ^51– 53^. But there is also evidence in subjects without dopamine deficiency: intravenous levodopa is rapidly taken up from the bloodstream into the brain and converted into dopamine, and several studies show that it then boosts synaptic dopamine release [reviewed in 12]. For instance, exogenous levodopa produces clear sedative and cognitive effects in healthy people ^54– 56^. Thus the authors originally expected that exogenous levodopa would decrease striatal RAC* binding.

However, further reflection and reading have motivated a different view whereby the results support the original goal of choosing a pharmacological challenge agent that would stimulate phasic dopamine release, but under endogenous control. Recall that the concern with stimulants as challenge agents was that they cause a substantial release of dopamine at the striatal synapse regardless of current environmental demands; this approach may produce a ceiling effect for dopamine release that does not reflect typical endogenous control. A sensible hypothesis to explain the results of the present study would be that a research subject lying awake in a quiet, darkened room without specific cognitive demands has no need for substantial phasic release of dopamine, and thus even if exogenous levodopa has added dopamine to presynaptic vesicles, they are not released at a substantial rate at the synapse. A levodopa-raclopride study of a motor task in healthy individuals provides direct experimental support of this hypothesis ^57^. That study was properly designed with two sessions, placebo on one day and levodopa on another, with randomized order. Levodopa increased striatal dopamine release during performance of a motor task, but not at rest. Since in the present study all subjects were at rest during all scans, the results are consistent with those of Flöel and colleagues ^57^.

### TS and control group comparisons

The tic and control subgroups have only five subjects each, and differences between the tic and control groups in the *a priori* VOIs were not statistically significant, so there is little need to comment further on these results. Previous drafts of this report included such discussion ^58^.

The whole-brain analysis comparing RAC* binding with levodopa vs. placebo did identify statistically significant differences ( Figure 8a–c). In midbrain (approximately substantia nigra/VTA) and in parahippocampal gyrus, levodopa stimulated dopamine release in controls but reduced it in TS subjects in. A similar pattern, though not statistically significant, was observed in orbital cortex (Brodmann’s area 13), thalamus and globus pallidus ( Figure 7 and Figure 8d).

One expects exogenous levodopa to increase dopamine release in the substantia nigra, as occurred in the control subjects. D _2_ and D _3_ dopamine receptors are present in the substantia nigra and their activation inhibits spike firing, dopamine synthesis and dopamine release by nigral dopaminergic cells ^59^. We hypothesize that levodopa increased dopamine stimulation of these inhibitory D2-like receptors in control subjects, and this may have prevented levodopa from stimulating nigrostriatal dopamine release into the striatum.

Subjects with TS, however, showed an increase in substantia nigra RAC* binding with levodopa, consistent with a decrease in nigral dopamine release. Nigral dopamine release has been related to reward and novelty in humans. Healthy adults with higher novelty seeking scores had lower D2-like binding ([ ^18^F]fallypride) in SN, consistent with greater dopamine release ^60^. Functional MRI studies have also demonstrated substantia nigra signal related to stimulus novelty or to the Novelty Seeking trait ^61– 63^. Healthy adults receiving a sweet vs salty taste had BOLD activation in this region ^64^. Despite this information, it is not clear how to relate a decrease in levodopa-stimulated dopamine release in substantia nigra to the pathophysiology of TS. Explaining the similar difference in nigral levodopa response in TS in parahippocampal gyrus and orbital cortex is no easier, though dopaminergic effects on D2-like binding in hippocampus have been documented in Parkinson disease ^65^ and dopamine agonists evoke changes in orbital cortex activity ^66^. The trend for a similar effect in thalamus is consistent with a [ ^11^C]FLB-457 PET study in which amphetamine provoked thalamic dopamine release in control subjects but not in TS ^67^. Overall, these results are consistent with an abnormality of presynaptic dopaminergic pharmacology in TS, but the limitations of this comparison must be acknowledged.

### Limitations

Higher-affinity radioligands, such as [ ^18^F]fallypride or [ ^11^C]FLB 457, have advantages for measuring cortical D2Rs, *e.g.* in the frontal lobe where D2Rs appear at much lower concentrations than in the striatum. There are two primary concerns with RAC* outside the striatum [reviewed thoroughly in 9]. The first concern is a reliability issue: since the concentration of D2-like receptors is low in cortex compared to striatum, the counting statistics are poor for cortical VOIs of similar volume, and this renders the computed BP _ND_s suspect. For instance, some regional RAC* BP _ND_s are negative or close enough to zero that displacement studies produce results that are hard to interpret. In the present study, FreeSurfer-defined cortical regions allowed the creation of a large, reliably defined frontal lobe VOI, in which PET time-activity curves were low in noise ( Figure 3), allowing statistically reliable estimates of BP _ND_ that were uniformly positive ( Table 4, Figure 4). Similarly RAC* displacement in thalamus has shown adequate counting statistics and reliability in previous studies ^47, 68^.

The second concern with RAC* in extrastriatal regions is one of validity or interpretation. RAC* binding in cortex includes some nonspecific binding ^33^, so a fair question is to what extent specific binding in cortex represents dopamine D2-like receptors. D2 and D4 receptors are expressed in human prefrontal cortex, though at relatively low concentrations compared to striatum ^69^. On the other hand, at least one study’s results suggest that raclopride may have superior sensitivity to fallypride for measuring dopamine release in some cortical regions ^70^. The validity concern is less worrisome in human thalamus, which contains predominantly D _3_ rather than D _2_ receptors ^71^, and in substantia nigra, where D _2_ and D _3_ receptors are well characterized. Other authors have interpreted substantia nigra RAC* displacement as indicating synaptic dopamine release ^9^.

Finally, comparing TS and control subgroups of only five subjects each provides insufficient power to identify some true group differences (type II error). More importantly, the small sample size lowers confidence in how representative the statistically significant differences are of the overall population of adults with TS.

### Future directions

These results suggest that a natural next step for research in TS is to test whether dopamine release in TS differs during a dopamine-releasing cognitive (or other) task. Levodopa may augment the task-evoked release or interact with it differently in people with versus without tics. Along these lines, a cognitive-pharmacological interaction fMRI study in TS found that LD changed the BOLD responses to a working memory task ^72^. A newer levodopa infusion method produced roughly twice as high a levodopa plasma concentration as the infusion used in this study ^12^, and may produce greater dopamine release.

PET images and clinical data The spreadsheet in OpenDocument file format provides the clinical data and links each PET scan to the subject scanned and the condition (i.e., before or during the placebo or levodopa infusion). Also 40 PET files are provided with the filename extension .v, one for each dynamic PET scan. These files are in ECAT file format; users of other imaging file formats will find useful information at the following web site: http://www.turkupetcentre.net/petanalysis/format_image_ecat.html

## Data availability

*F1000Research*: Dataset 1. PET images and clinical data, 10.5256/f1000research.5672.d42172 ^74^

## Consent

All subjects provided written confirmation of informed consent before study participation.
