# Supplementary material for: Levodopa effects on [ 11C]raclopride binding in the resting human brain
Source: F1000Res. 2015 Jan 23;4:23. [Version 1] doi: 10.12688/f1000research.5672.1 (PMC4490799; doi:10.12688/f1000research.5672.1)
Supplement: Peer review history

# Peer review: Referee response for version 1

**DOI:** 10.5256/f1000research.6062.r8060

**Published:** 2015-03-23

**Authors:** Lars Nyberg

**Affiliations:** 1 Umeå Center for Functional Brain Imaging, Umeå, Sweden

The most novel aspect of the study was the investigation of levodopa. This is very interesting. No significant levodopa effects were, however, observed. The authors offer some interesting thoughts on the reason for this null effect, most critically pointing to a need to have an active task PET design. This is a plausible argument that may stimulate further research on this topic.

A potentially interesting methodological contribution is the observation of a difference between the 1 ^st^ and 2 ^nd^ scan on each day of scanning.

The study is likely underpowered, in particular for any group comparison (5 TS, 5 ctrls), so the repeated-measures analysis was most likely not very sensitive. I would treat the observed differences between TS and control groups from the whole-brain analysis as preliminary.

I may have missed it, but I could not find information about how the PET scans were reconstructed.

I have read this submission. I believe that I have an appropriate level of expertise to confirm that it is of an acceptable scientific standard.

# Response

**Published:** 2015-03-24

**Authors:** Kevin J Black

**Affiliations:** Department of Psychiatry, Washington University in St. Louis, USA

The authors thank Prof. Nyberg for the thoughtful review. 

The [ ^11^C]raclopride PET data were collected in 3D mode and reconstructed on the Siemens ECAT 961 scanner console using filtered backprojection (ramp filter), with attenuation measured before each emission scan using an external ^68^Ge/ ^68^Ga source.

We agree with all the reviewers that the between-group comparison is useful primarily as pilot data because of the small group sizes.

# Peer review: Referee response for version 1

**DOI:** 10.5256/f1000research.6062.r7821

**Published:** 2015-03-23

**Authors:** W.R. Wayne Martin

**Affiliations:** 1 Division of Neurology, University of Alberta, Edmonton, AB, Canada

This is a carefully performed study that presents a novel approach to measure presynaptic dopamine release using the administration of exogenous levodopa, coupled with raclopride PET scanning. Preliminary data are provided using this method in a small group of controls and subjects with Tourette syndrome. 

The authors describe a decline in dopamine release in striatum, thalamus and frontal lobe between the first and second scan of each day in response to placebo administration in normal subjects, possibly due to habituation to study procedures. Levodopa administration did not alter striatal dopamine release differently in Tourette syndrome vs. controls. However, dopamine release differed significantly in the midbrain and parahippocampal gyrus in the two conditions. Levodopa stimulated dopamine release in controls but reduced it in Tourette subjects.

Although these are important observations, the number of subjects studied was small. Hence, these must be considered pilot data although they are consistent with a rather complex dopaminergic role in Tourette syndrome. Of interest for future studies would be the evaluation of task-evoked dopamine release in response to cognitive tasks. Lastly, the observation that habituation occurs in response to placebo infusions has important implications to the interpretation of placebo-controlled studies of dopamine release.

I have read this submission. I believe that I have an appropriate level of expertise to confirm that it is of an acceptable scientific standard.

# Peer review: Referee response for version 1

**DOI:** 10.5256/f1000research.6062.r7601

**Published:** 2015-03-17

**Authors:** Marie Vidailhet

**Affiliations:** 1 Department of Research of Technology and Development, Hôpital Pitié Salpêtrière, Paris, France

The authors studies the raclopride binding (displacement) in groups of subjects (normal and Tourette syndrome). They studied the effect of levodopa infusions and of a placebo. The subjects were studied at rest.

Basically, they found that in Tourette syndrome, dopamine release was smaller  (reduced) than in controls, in midbrain (approximately substantia nigra/VTA) and in parahippocampal gyrus.

This is an interesting paper and the methodology is adequate. The subjects are studied at rest, this may underestimate the dynamic of dopamine release and it would have be more interesting to study this phenomenon during a task. The groups are very small, and the effect in Tourette syndrome may also be different according to the characteristics of the patient (with or without additional behavioral disorders).

Nevetherless, the study is consistent with the presence of abnormality of presynaptic dopaminergic pharmacology in Tourette syndrome.

I have read this submission. I believe that I have an appropriate level of expertise to confirm that it is of an acceptable scientific standard.
